# Novel Mode of Controlling Hemorrhage

**Published:** 1871-05

**Authors:** 


Novel Mode of Controlling Hemorrhage.—Dr. J. H. H. Burge (*N. Y. Med. Jour.*) contributes the history of a case of Placenta Prævia attended with profuse hemorrhage. He found a small margin of the placenta presenting. The liquor amnii had completely drained away, so that the outline of the child could be made out through the abdominal walls. The examination excited some pain and caused considerable hemorrhage. Observing that during the pain the pressure of the child's head upon the placenta controlled the flow, he in the intermission grasped the uterine tumor and pressed steadily in the direction of the os. This was continued with perfect success for two and a half hours, when a living child was born, and the placenta followed without loss of time. During all this time, if the pressure was relaxed while the uterus was inactive, hemorrhage was sure to follow.—*Medical Record.*

